# Causes of chronic pain unrelated to surgical trauma after groin hernia repair: a prospective cohort study

**DOI:** 10.1007/s10029-024-03201-x

**Published:** 2024-11-16

**Authors:** Lovisa Kroon, Kristina Ticehurst, Jukka Ahonen, Jonny Norrby, Fernando Ruiz-Jasbon

**Affiliations:** 1 Department of Surgery, Halland’s Hospital, Tölövägen 5, Kungsbacka, 434 80 Sweden; 2 Department of Surgery, Institute of Clinical Science, Sahlgrenska Academy at University of Gothenburg, Gothenburg, Sweden; 3 CEO Capio Gastro Center, Gothenburg, Sweden

**Keywords:** Chronic pain, Inguinal hernia, Groin hernia, Chronic post-surgical pain, Pain questionnaire, Surgical trauma, Pain assessment

## Abstract

**Background:**

Chronic inguinal pain (CIP) can be caused by musculoskeletal or neurological pathologies and by surgical trauma after inguinal hernia repair among other. The aim of this prospective cohort observational study was to find the incidence and causes of CIP unrelated to surgical trauma 12 months after inguinal hernia repair.

**Methods:**

During sixteen months patients consulting a hernia center for groin-related symptoms were included in the study. Patients were evaluated by surgeons and filled out preoperatively the Inguinal Pain Questionnaires and a Numerical Rating Scale pain-questionnaire. For patients undergoing inguinal hernia repair, postoperative questionnaires similar to the pre-operative ones were sent out at 12 months. Patients scoring pain on pain questionnaires were evaluated by phone and physical examination.

**Results:**

289 patients (78.1%) of 370 repaired patients filled in the postoperative questionnaires. 62 (21.4%) patients scored pain, of these patients 5 (1.7%, 5/289) answered incorrectly in the pain questionnaires and 14 (4.8%, 14/289) had non-surgical trauma causes of pain: 5 musculoskeletal, 4 neurological and 3 other medical pathologies.

**Conclusions:**

This cohort study found CIP unrelated to surgical trauma in 4.8% of patients undergoing a groin hernia repair. Most causes of pain unrelated to surgical trauma were musculoskeletal and neurological pathologies. Nearly a third of patients scoring inguinal pain on pain-questionnaires did not have chronic post-surgical pain (CPSP), therefore incidence of CPSP should not be based solely on pain questionnaires. Clinical assessment of patients with pain is necessary to excluded CIP unrelated to the surgical trauma.

**Supplementary Information:**

The online version contains supplementary material available at 10.1007/s10029-024-03201-x.

## Introduction

Chronic inguinal pain (CIP) can be caused by different pathologies. These include among other: muscular disruptions after physical activities, skeletal changes such as coxarthrosis or femoral-acetabular impingement (FAI), primary entrapment of nerves in the zone, secondary to groin or incisional hernias or by surgical trauma such as inguinal hernia repair or hip arthroplasty [1–5]. The last two causes of chronic inguinal pain are often mentioned in the literature as Chronic Post-surgical Pain (CPSP) [5].

There is no consensus regarding how chronic post-surgical pain after inguinal hernia repair is defined, measured and evaluated. This creates significant heterogeneity in rates of chronic post-surgical pain (2-63%) making comparability between studies extremely challenging [6].

Physicians working at hernia centers rarely treat patients with chronic post-surgical pain on a daily basis. This is illustrated by the low rate of reoperation due to chronic post-surgical pain (< 0.1%) according to the Swedish Hernia Register. However, in the same register the rate of chronic post-surgical pain is estimated to nearly 16% when assessed solely by a post-operative pain questionnaire [7, 8]. Surely if a procedure entailed such a high rate of chronic post-surgical pain, efforts should be made to achieve a better understanding of this incongruence between questionnaire-based rates of chronic post-surgical pain and the “perception” of many hernia surgeons on the frequency of chronic post-surgical pain. It is possible that questionnaire-based studies over-diagnose chronic post-surgical pain or include patients with other causes of chronic inguinal pain. Contrarily, it is also possible that patients with chronic post-surgical pain do not consult the same surgeon or clinic that performed the primary operation. Regardless, considering the routine application of inguinal hernia surgery, any complication poses a significant burden to global health care.

Even though there is copious literature on chronic post-surgical pain after hernia repair, to our knowledge there is no previous study that specifically evaluates non-surgical causes of chronic inguinal pain after hernia repair that include an assessment of pain and clinical examination both pre- and post-operatively.

The present study constitutes a segment of a broader research project exploring inguinal pain and inguinal hernia symptoms. The general project intends to investigate various aspects of primary inguinal pain in patients with and without hernias, risk factors and treatment of chronic post-surgical pain, assessment methodologies for chronic post-surgical pain, the influence of hernia repair on pre-operative symptoms, and the causes of chronic pain unrelated to surgical trauma following hernia repair.

### Aim

The primary endpoint of this study is to find the incidence and causes of chronic inguinal pain unrelated to surgical trauma 12 months after inguinal hernia repair.

## Methods

This prospective cohort observational study was conducted at the Department of Surgery at Hallands Hospital /Kungsbacka, Sweden. The clinic is an outpatient hernia center with experienced surgeons in both laparo-endoscopic and open mesh repairs. The clinic covers more than 80% of all planned hernia repairs of the Region Halland with 320 000 inhabitants including hernia surgery training of all trainee surgeons of the region.

Between June 2018 to September 2019, all adult patients consulting the clinic for groin-related symptoms were asked to participate in the study. Patients unwilling to join the study or that unable to understand or fill in the written questionnaires were not included.

The study was approved by the regional ethics committee (Protocol 2018/2, section “Methods”).

### Assessment of pain and quality of life

Patients included in the study filled out a preoperative questionnaire and were clinically evaluated by a surgeon according to a checklist. The checklist included an evaluation of sensitivity to touch and pain on palpation of the inguinal region, lower abdomen, genital region and proximal part of the thigh, as well as evaluation of the limitations in range of motion or pain on passive and active mobilization of the hip. The complete checklist is in the supplementary material. The preoperative questionnaire included two validated pain and quality of life questionnaires: Inguinal Pain Questionnaire (IPQ) and Numerical Rating Scale 0–10 (NRS) at different activities [9–11]. Patients with bilateral hernias filled in a questionnaire for each side thus they are counted twice.

For patients undergoing inguinal hernia surgery, perioperative data were recorded in the Swedish Hernia Registry. A postoperative questionnaire similar to the pre-operative one was sent out at 12 months to assess patients´ pain, Quality of Life (QoL) and the effect of the hernia repair on pre-operative symptoms.

At 12 months follow up, patients experiencing discomfort or pain in the questionnaire were contacted by a physician. Primarily these patients were evaluated by phone consultation and offered a physical evaluation similar to the pre-operative one. Both assessments were done using a checklist (supplementary material). In cases where only surgeons performed the evaluations, these were done on different appointments. If necessary, other specialists such as orthopedist or anesthesiologist were consulted.

### Type of hernia repair

The type of hernia repair was decided in agreement between a consultant surgeon and the patient in the pre-operative consultation. All patients were operated electively. All consultant surgeons of the clinic were experienced in both laparo-endoscopic and open mesh repairs. The majority of patients were offered a modified Lichtenstein repair with a lightweight polypropylene self-griping mesh or a Totally Pre-peritoneal (TEP) repair with a large pre-formed heavy-weight polypropylene mesh without fixation. However, in some clinical situations an open pre-peritoneal mesh repair with a preformed lightweight polypropylene mesh or a Trans-abdominal pre-peritoneal (TAPP) with a large pre-formed heavy-weight polypropylene mesh without fixation could be offered.

### Definition of chronic postsurgical pain and chronic pain unrelated to surgical trauma after hernia repair

The study uses the International Association for the Study of Pain (IASP) definitions of chronic pain and chronic postsurgical pain [5, 12, 13].

*Chronic inguinal pain (CIP)* is defined as inguinal pain that persists or recurs for longer than 3 months; this study means NRS ≥ 1 at any activity or any type of pain in IPQ.

“Chronic post-surgical pain *after hernia repair*” (CPSP) is defined as chronic inguinal pain that *develops* or *increases* after the repair; persists at least 3 months after the surgery; the pain is localized to the surgical field or is projected to the innervation territory of a nerve situated in this area and excluding other causes of pain, i.e. pre-existing pain. This definition means that the traumatic etiology of the pain should be highly probable. The International Association for the Study of Pain does not include hernia recurrence, pre-existing pain and other chronic pain unrelated to surgical trauma as chronic post-surgical pain.

*“Chronic pain unrelated to surgical trauma after hernia repair*” is defined as all causes of chronic inguinal pain excepting chronic post-surgical pain.

In order to classify the pain as related (CPSP) or unrelated to surgical trauma, two physicians had to separately come to the same conclusion, including the cause of pain.

### Statistics and sample size calculation

Based on estimates from the Swedish Hernia Register, the study calculated that approximately 16% of patients would experience pain one year after hernia repair. Thus, the study determined that a sample of 350 hernia-repaired patients would be needed, allowing for a 10% dropout rate, to identify at least 50 patients reporting inguinal pain after hernia repair. Given that the clinic typically conducts between 300 and 500 hernia repairs annually, it was calculated that at least one year would be required to achieve the number of patients with inguinal pain necessary for this study.

Data were analyzed by descriptive statistic (SPSS™ statistic 24).

## Results

During the study period 620 patients were screened for eligibility in the study. 574 patients were included, and 370 (64.4%) patients underwent hernia repair. Mean age was 63.8 (25–88 year), 94% were male, 27% were bilateral repairs. All patients were repaired with mesh: 52.2% using open repair and 47.7% laparo-endoscopic, other perioperative data are shown in Table 1. After 12 months 289 patients (78.1%) filled in postoperative questionnaires. The median time between the date of filling out the postoperative questionnaires until the first telephone evaluation of the pain was 1.5 months (0–10 SD 2.6) and until the physical evaluation of the cause of the pain 4 months (0–48 SD 8.4).

**Table 1:** Preoperative and intraoperative data

Age	Mean years (min-max)	63.8 (25–88)
Body Mass Index	Mean kg/m^2^ (min-max)	25.2 (17.7–52.6)
Gender	Female: number (%)	22 (5.9)
	Male: number (%)	348 (94.1)
Hernia Type	Primary: number (%)	348 (94.1)
	Recurrent: number (%)	22 (5.9)
	Unilateral: number (%)	270 (72.9)
	Bilateral: number (%)	100 (27.0)
Hernia Repair	Lichtenstein* number (%)	176 (47.6)
	Open preperitoneal number (%)	17 (4.6)
	TEP number (%)	166 (44.9)
	TAPP number (%)	11 (3.0)
Hernia Classification	Lateral: number (%)	191 (52.3)
	Medial: number (%)	119 (32.6)
	Femoral: number (%)	17 (4.7)
	Combined: number (%)	34 (9.3)
	Funicular lipoma: number (%)	4 (1.1)
Operative Time	Mean minutes (min-max)	56.1 (23–93)

*Modified using self-gripping mesh. TEP: Totally Extra Peritoneal (Endoscopic)

TAPP: Transabdominal Preperitoneal (Laparoscopic)

The mean pain score of the cohort during activities decreased from NRS 1.97 (SD 2.4) (Min 0, Max 10) preoperatively to 0.40 (SD 1.3) (Min 0, Max 10) postoperatively.

**Fig. 1:**
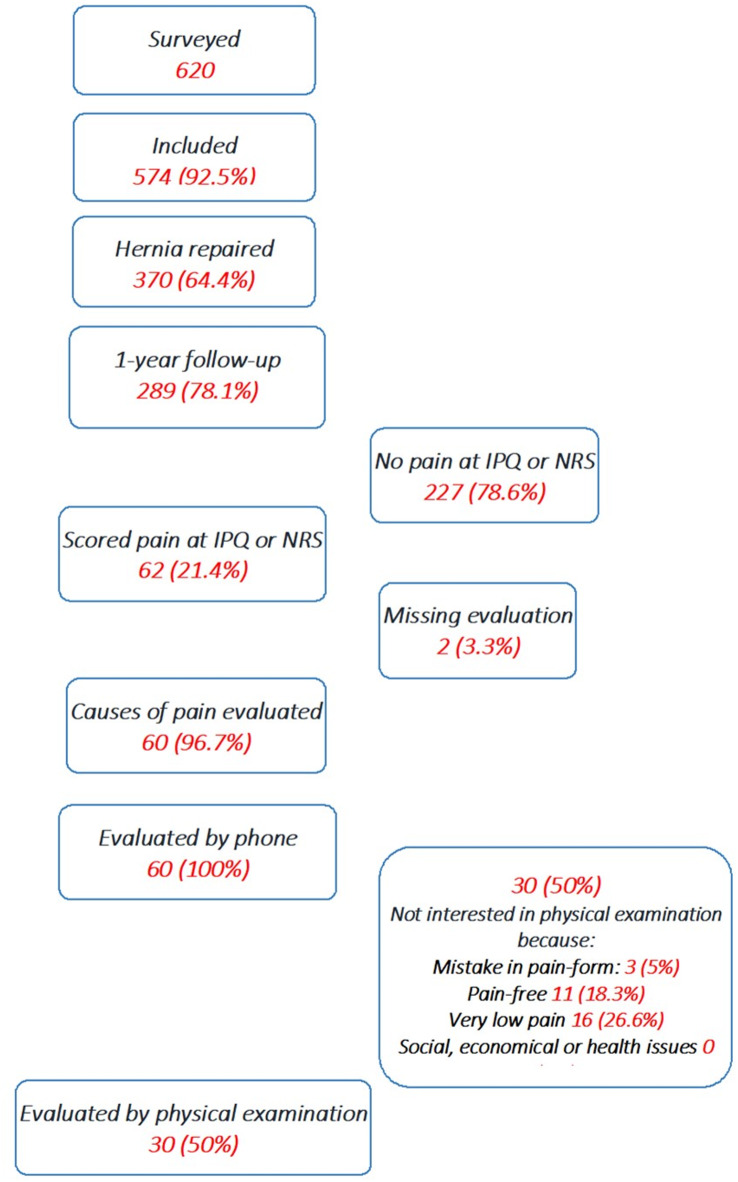
Flow of the patients during the study

62 patients (21.4%) stated discomfort or pain one year after surgery, 19 patients scored NRS ≥ 3. The mean NRS of these 62 patients was 1.9 (SD 2.3) (Min 1-Max 10). Of these, 60 (96.7%) were evaluated by phone consultation and offered a physical evaluation. 30 (50%, 30/60) patients accepted a physical examination. The main reasons for not attending a physical examination of the remaining 30 (50%, 30/60) patients were absence or improvement of pain in such a way that patients did not consider a medical examination necessary (Fig. 1). Both patients who could not be contacted by phone rated pain 2 or less on NRS.

### Patients with chronic pain unrelated to surgical trauma

**Fig. 2:**
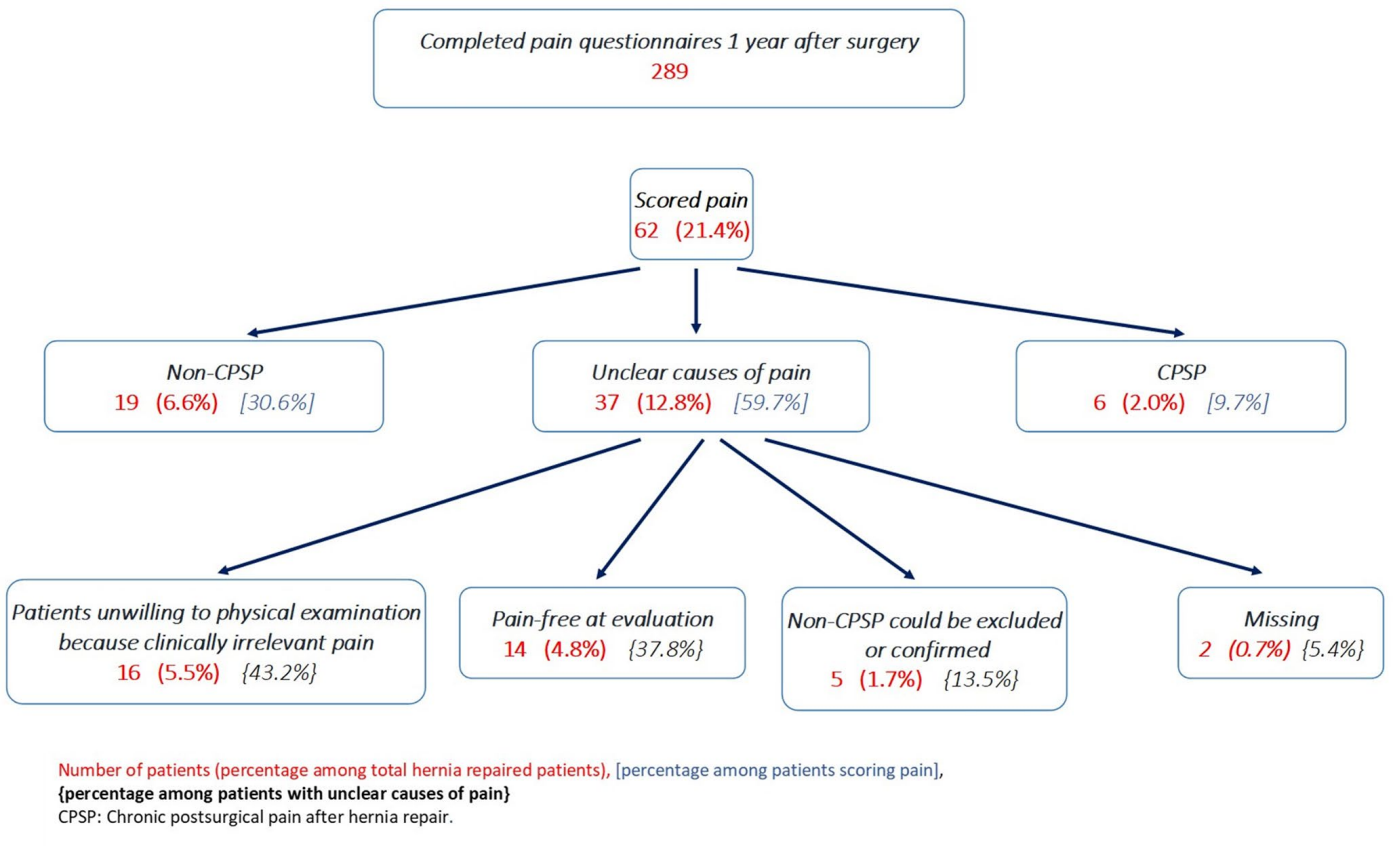
Rate of the different causes of inguinal pain at 1 year after hernia repair

Nineteen of the sixty-two patients (30.6%, 19/62) scoring pain after surgery had non chronic post-surgical pain according to two different specialists, it means 6.5% (19/289) of the postoperative assessed patients. 14 (4.8%, 14/289) patients had chronic pain unrelated to surgical trauma and 5 (1.7%, 5/289) patients answered incorrectly in the pain questionnaire (Fig. 2).

There was a variety of causes of chronic pain unrelated to surgical trauma: musculoskeletal 35.7% (5/14), neurological pain 28.5% (4/14), gynecological or urological 14.2% (2/14), hernia recurrence or funicular lipoma 14.2% (2/14), and other medical issues 7.1% (1/14). (Table 2).

In the group of patients with chronic pain unrelated to surgical trauma, the mean of the maximum NRS at any activity was 4.7 before the surgery and 4.3 one year after the hernia repair. Data about NRS or IPQ values and the cause of pain in these patients shows in Table 2.

**Table 2:** Patients with chronic pain unrelated to surgical trauma after hernia repair

Sex, age (years)	Cause of Pain	NRS Pre Post		IPQ Pre Post		Specialists evaluating pain	Type of hernia*	Surgery
Male, 86	Spinal Stenosis L2-L3, L3-L4	3	10	3	6	Ort, Int, Surg	F1 R	Lichtenstein + stiches on femoral canal
Male, 70	Coxarthrosis	5	7	4	4	Ort, Surg	M3 P	TIPP
Male, 68	Coxarthrosis	7	1	3	2	Ort, Surg	L2 R	TEP
Male, 68	Hip prosthesis related	1	2	2	3	Ort, Surg, GP	L1 P	Lichtenstein
Male, 65	Peripheral neuropathy near umbilicus	2	4	4	4	An, Surg, Ort	L2 P	Lichtenstein
Male, 57	Prostatitis	7	4	6	3	Uro, Surg	L1 P, lipoma	Lichtenstein
Male, 53	Inguinal hernia recurrence lateral	0	3	1	3	Surg, Surg	F1 P,	TEP, bilateral
Male, 49	Osteitis Pubis	6	2	5	4	Ort, Surg	M2 P	TEP
Male, 46	Herniated Disk L2-L3, L5-S1	9	6	7	4	Ort, Surg, Neur, GP	F1 P	TEP
Female, 45	Ligamentum Rotundum lipoma	1	1	2	2	Surg, Surg	F1 P	TEP bilateral
Male, 43	Neuropathy ilioinguinal nerve as preoperatively	5	8	6	3	An, Surg, Ort	0 P, lipoma	TEP with self-gripping mesh, bilateral
Female, 43	Dysmenorrhoea	9	4	6	3	Gyn, Surg	F1 P	TEP
Male, 36	Abdominal muscular pain as pre-operatively	5	2	4	4	GP, Surg	L2 P	TEP
Female, 27	IBS	6	6	6	4	GP, Surg	L1 P	TEP

Ort: Orthopedist, Int: internist, Surg: surgeon, GP: general practitioner, An: Anesthetist, Uro: urologist, Neur: Neurologist, Gyn: Gynecologist, *: European Hernia Classification; P: primary, R: recurrent, L: lateral, M: medial, F: femoral, 1: <1.5 cm, 2:<3 cm, 3: ≥3, Lichtenstein: Modified Lichtenstein with selfgriping mesh, TIPP: transinguinal preperitoneal with light mesh without stiches, TEP: Totally extraperitoneal with preformed mesh without fixation. NRS: Numerical Rating Scale (0–10) of the maximum pain at any activity. IPQ: Inguinal Pain Questionnaire; (1) no pain. (2) easily ignored. (3) cannot be ignored but does not affect daily activities. (4) cannot be ignored and affects some activities. (5) Restrict performing most activities (6) Requires long periods of rest. (7) Required immediate medical attention

### Patients with chronic post-surgical pain or unclear cause of pain

Six patients (2%, 6/289) had chronic post-surgical pain according to two different specialists and 37 (12.8%, 37/289) had unclear cause of pain (Fig. 2).

In the group “unclear cause of pain” 2 (5.4%, 2/37) patients were missing and 27 (72.9%, 27/37) patients could not be physically evaluated. The reason for absence of physical examination was patients` lack of interest because of the modest symptomatology: 11 patients were pain free and 16 patients thought the pain was not an issue for them. All 29 patients, including missing patients, rated pain at rest < 3 on NRS. The remaining 8 (21.6%, 8/37) patients were evaluated by several specialists. Despite multidisciplinary evaluation, chronic post-surgical pain or chronic inguinal pain unrelated to surgical trauma could not be confirmed or excluded, 3 of those 8 patients became pain-free during medical investigation (Fig. 2).

## Discussion

This study found that nearly a third of the hernia repaired patients that scored discomfort or pain on two validated pain questionnaires at 1-year follow-up did not have chronic post-surgical pain (CPSP) according to the International Association for the Study of Pain definition. More specifically this means that nearly 5% of all hernia repaired patients had chronic pain unrelated to surgical trauma one year after the surgery, in addition 1.7% scored pain by mistake on post-operative pain questionnaires. This knowledge is of great importance for the surgeon when assessing patients with postoperative chronic pain.

### Musculoskeletal disorders as cause of pain after hernia repair

Musculoskeletal disorders were the most common cause of chronic pain unrelated to surgical trauma in this study. 5 patients in this study had musculoskeletal disorders as cause of pain, 3 of these related to hip-osteoarthritis or hip-prosthesis problems. In all these patients the conclusive diagnosis was made by an orthopedist after radiological and physical examination and one patient received a new prosthesis because of the diagnosis. One patient had osteitis pubis confirmed by radiology and orthopedist examination and in the last patient in the group of musculoskeletal disorders the pain was identical to the pain at exercise in the obliquus muscle as before the hernia repair (TEP). According the IASP the pain must change or increase after the surgery in order to judge the pain as post-surgical pain.

Epidemiological and cohort studies have shown that pain related to musculoskeletal pathology is common in the adult population. The prevalence of hip pain and coxarthrosis can be as high as 19.2% and 9.7% respectively in persons older than 60 years of age and the prevalence increases with age [14–16].

The fact that the prevalence and incidence of groin hernias are also highest in people between 60 and 85 years of age means that those patients have a higher risk of chronic inguinal pain related to musculoskeletal pathologies too [17, 18]. Furthermore, the risk of chronic inguinal pain being secondary to musculoskeletal disorders can increase with longer follow up after groin hernia surgery because patients become older and therefore have a higher prevalence of musculoskeletal pathologies. In other words, a 3-year follow up after hernia repair might include more patients with musculoskeletal chronic inguinal pain than a 1-year follow-up.

### Neuropathic disorders as cause of pain after hernia repair

Neuropathic pain conditions not associated with hernia repair in this study were lumbar radiculopathy and peripheral nerve pain. This represents a rate of 1.4% (4/289) of the cohort of hernia-repaired patients. In the general population the incidence of lumbar radiculopathy is nearly 3–5% and it increases with age reaching a rate of 10% after 40 years of age [1, 19, 20]. L1 or L2 radiculopathy can be presented with just pain in the groin or proximal thigh without backpain which makes diagnosis difficult [20].

Peripheral neuropathy in the abdomen or groin is probably less common than radiculopathy in the general population. Peripheral neuropathy near the groin can be difficult to differentiate from inguinal hernia symptoms and consequently leading to a hernia repair in patients with incipient groin hernia without real hernia symptoms [21]. This could explain why patients with a hernia only diagnosed by ultrasound and atypical symptoms have a higher risk of chronic inguinal pain after hernia repair [22].

### Hernia recurrence as cause of pain after hernia repair

Hernia recurrence is not considered as cause of chronic post-surgical pain according to International Association for the Study of Pain because the origin of pain is not surgical trauma but an insufficient repair. Both chronic post-surgical pain and recurrence are the most important outcomes after hernia repair. However, the surgical treatment of chronic post-surgical pain after hernia repair is contrary to the treatment of inguinal hernia recurrence: removal of the mesh with peripheral neurectomy versus implantation of a new mesh and nerve conservation. Another reason to separate pain related to recurrence from chronic post-surgical pain is that accuracy and scientific value of intervention studies can change if the results do not separate painful recurrence from chronic post-surgical pain. Consider a scenario where a randomized study compares two types of meshes. Mesh A demonstrates a 1% rate of painful recurrence and a 4% rate of chronic post-surgical pain, while Mesh B exhibits the opposite trend. If the study were to classify painful recurrence as chronic post-surgical pain, it would overlook significant differences between the meshes. However, by distinguishing between painful recurrence and chronic post-surgical pain, the study would reveal that Mesh A is preferable for patients with a higher risk of recurrence and a lower risk of chronic post-surgical pain, whereas Mesh B is more suitable for patients with higher risk of chronic post-surgical pain and lower risk of recurrence.

### Other non-surgical causes of pain and errors in filling out the pain-form after hernia repair

The number and the percentage of patients with medical, urological, gynecological causes of pain or filling-errors in the pain-forms were very low in relation to the total cohort if each cause is observed separately, between 1 case (0.3%, 1/289)) and 5 cases (1.7%, 5/289). However, all these other causes together represent 12.9% (8/62) of the patients scoring pain in the questionnaire. It means that assessment of the causes of pain after hernia repair should be done not solely by surgeons or “pain specialists”. Several specialists should be included in the assessment of the pain in order to get a higher accuracy of the causes of chronic inguinal pain. The diversity of the causes of chronic inguinal pain is a further reason for why assessment of chronic post-surgical pain after hernia repair with solely pain-questionnaires without physical evaluation has a low scientific value.

### Concordance and difference of the present study with the literature

Our results show the same trend as previous studies that included postoperative clinical examination in patients scoring pain on pain-questionnaires after hernia repair if those studies had used the International Association for the Study of Pain definition of chronic post-surgical pain (CPSP) as in the present study [23, 24].

Haapaniemi et al. found 33% non-CPSP in patients with pain 3 years after hernia repair: 18% recurrence, 5% of hip pathologies, 5% back pain, 2.5% urogenital pathologies and 2.5% IBS. Kalliomäki et al. found 30% non-CPSP at examination in patients scoring pain in a questionnaire 4.6 years (mean) after hernia repair: 22% were pain free at examination, 4% had a recurrence, lower back pain was found in 2% and others causes in 2%.

Both above mentioned studies classified all patients with “neuropathic pain” and “musculotendinous/ muscular nociceptive pain” at physical examination as chronic post-surgical pain. Haapaniemi included all patients with “poorly localized pain in the groin area exacerbated by voluntary contraction” in the group of “musculotendinous pain”. Kalliomäki classified all patients with “muscular nociceptive pain” or “difficult classified pain” between the umbilicus and 20 cm beyond the groin on the thigh as chronic post-surgical pain. Both studies lacked a physical examination and pain assessment preoperatively. Therefore, these studies classified even patients who already pre-operatively had neuropathic, muscular, or difficult classified pain in the groin or lower abdomen as chronic post-surgical pain. In other words, patients with muscular and neuropathic pain unrelated to hernia repair could be incorrectly classified as chronic post-surgical pain in these studies.

Another difference of the present study with the previously mentioned literature is who assessed and classified the chronic inguinal pain as non-CPSP. In the study of Haapaniemi it was a surgeon and in the Kalliomäki study it was mostly an anesthesiologist (only 16% were examined by a surgeon). In the current study all patients classified as non-CPSP were examined by at least 2 different specialists and if necessary radiological evaluation was included. Surgeons, anesthesiologists, orthopedists, gynecologists, general practitioners, urologists and internists were included in the evaluation if necessary and these specialists initiated treatment according to the identified cause of the pain. Thus, the accuracy of assessment of non-surgical related pain in this study was higher than in previous studies reducing the risk of observer or confirmation bias.

### Limitation and strength of the study

A limitation of the study is the large percentage of patients with “unclear cause of pain”, 59.7% of the patients who scored pain or 12.8% of all the hernia-repaired patients. All patients in this study that confirmed pain at phone call and had phone consultation alone, were classified as having ”unclear cause of pain”. Patients that were physically examined were classified as having “unclear cause of pain” if the clinical assessment wasn’t sufficient to determine the cause of pain. It is very difficult in many cases to differentiate whether the pain is musculoskeletal/neuropathic or related to the surgical trauma and a clinical assessment is not a guarantee to identify the cause of the pain. However, the majority of patients included in this group (81%) no longer had pain or the patient considered the pain insignificant at follow-up. A confirmation of the low clinical value of the pain for these patients was that no-one rated the pain higher than 3 at NRS. Another cause of the high percentage of patients with “unclear cause of pain” was the strict criterium of a minimum of 2 different specialists agreeing on the cause of the pain. The fact that the origin of pain could not be addressed in patients scoring less pain highlights the difficulties to assess the cause of pain in this group of patients. However, patients scoring low pain are clinically less relevant.

The strengths of the present study are the multifactorial and longitudinal assessment of pain both preoperatively and postoperatively including physical examination and treatment by several specialists in addition to pain and quality of life questionnaires. This type of assessment permits a better accuracy of the classification of pain after hernia repair as chronic post-surgical pain (CPSP) or non-CPSP.

### External validity

The study was conducted at a hernia specialized clinic that takes care of the absolute majority of patients with inguinal hernia symptoms in the region. This implies that the population consulting the clinic was not selected. The staff consists of experienced hernia surgeons in both open and laparo-endoscopic mesh hernia repair. Therefore, it is likely that the percentage of patients with preoperative inguinal pain not related to the hernia and selected for hernia surgery could be lower compared to less specialized clinics. For the same reason, it is possible that the percentage of patients with chronic post-surgical pain could be lower compared to less specialized clinics [25]. It is therefore possible that a similar study in clinics not specialized in inguinal hernia surgery would have a higher percentage of patients with chronic post-surgical pain and patients with inguinal pain unrelated to surgical trauma.

## Conclusions

This cohort study found chronic inguinal pain unrelated to surgical trauma in 4.8% of patients undergoing a groin hernia repair and 1.7% of patients had filling-errors in the pain-questionnaires. Most causes of pain unrelated to surgical trauma were musculoskeletal and neurological pathologies but several other causes explained the pain. Nearly a third of patients scoring inguinal pain on pain-questionnaires did not have chronic post-surgical pain according to the definition of the International Association for the Study of Pain, therefore incidence of chronic post-surgical pain based solely on pain questionnaires has low accuracy. Clinical assessment is crucial to exclude chronic inguinal pain unrelated to the surgical trauma but it is not a guarantee to identify the cause of the pain in all patients.

## Electronic Supplementary Material

Below is the link to the electronic supplementary material.

Supplementary Material 1

Supplementary Material 2

## Data Availability

The authors would share the anonymized data of this article on justifiable request to the corresponding author.

## Declarations

### Previous presentation of the study

Part of the paper has previously been presented as abstracts at the EHS-AHS joint congress Hernia 2021 in Copenhagen, at the EHS-2022 congress in Manchester, at the AHS-22 congress in Charlotte, USA and at the BJS prize of the EHS-24 congress in Prag, therefore the abstract was published as a supplement of BJS (10.1093/bjs/znae122.001). The original paper has not been published and will not be simultaneously submitted or published elsewhere.
