# Supplementary material for: Causes of chronic pain unrelated to surgical trauma after groin hernia repair: a prospective cohort study
Source: Hernia. 2024 Nov 16;29(1):13. doi: 10.1007/s10029-024-03201-x (PMC11568985; doi:10.1007/s10029-024-03201-x)
Supplement: Supplementary file 1 — Supplementary Material 1 [file 10029_2024_3201_MOESM1_ESM.pdf]

Preoperative patient questionnaire regarding inguinal pain and inguinal hernia
Date
Swedish social security number
Name
In case of bilateral hernia, the questionnaire is filled out twice. This questionnaire is for: right, left

Tick the box that best resembles how you feel before possible surgery. 1 = strongly disagree, 5 = strongly agree.

1. I feel worried about general/local anesthesia.
2. I continuously think about general/local anesthesia.
3. I would like to know as much as possible about general/local anesthesia.
4. I feel worried about the surgery.
5. I think about the surgery continuously.
6. I would like to know as much as possible about the surgery.
7. Do you perceive pain in the thigh, hip or pelvic region on the same side as your presumed hernia?
8. Do you have pain in other areas of your body such as prostate, uterus, adjoining muscle?
9. Have you previously had surgery in the lower part of your abdomen/hip on the same side as your presumed hernia?
    a. If yes, what? Inguinal hernia, c-section, prostate, sterilization, other?

In case of bilateral hernia, please observe for the following questions only regard the side you ticked in the beginning of the questionnaire.

10. Have you observed a bulge in the inguinal region sometime the last month?
11. Do you perceive discomfort/pain in your genital region on the same side as the presumed hernia sometime over the last month?
    a. If yes, what? Pain, other.
    b. If other, please specify.
12. Have you had any inguinal discomfort/pain sometime during the last moth?
    a. If yes, what? Pain, other.
    b. If other, please specify.

If your answer to question 12 is that you have not experienced any discomfort the last month you are now finished with the questionnaire.
If you have not noticed a bulge in the inguinal region (question 10) skip to question nr 17.

13. Do you sometimes experience genital discomfort/pain on the same side as the presumed hernia when the inguinal bulge is present?
    a. If yes what complaint? Pain, other.
    b. If other, please specify.
14. If you experience genital discomfort, does it disappear or significantly decrease if you push the bulge back or if the bulge spontaneously goes back (i.e., if you lie down)?
    a. Yes, no, no genital discomfort.
15. Do you experience any inguinal pain or discomfort when the bulge is present?
    a. Yes, no
    b. If yes, what complaint? Pain, other.
    c. If other, please specify.
16. Does the inguinal discomfort/pain decrease significantly if you push the inguinal bulge back or if the bulge spontaneously goes back (i.e., if you lie down)?
    a. Yes, no
    b. If yes, what complaint decreases? Pain, other.

    c. If other, please specify.
17. If you experience inguinal/genital discomfort/pain, does it decrease if you press your hand against your inguinal region on the affected side?
    a. Yes, no, don't know.
18. Please grade your inguinal pain on the side of your presumed hernia when it was at its worst the last month:
    a. 1. no pain.
    b. 2. pain that is easily ignored.
    c. 3. pain that cannot be ignored but does not affect daily activities.
    d. 4. Pain that cannot be ignored and that affects concentration/daily activities
    e. 5. Pain that prevents performing most activities
    f. 6. Pain that requires long periods of rest/bedrest
    g. 7. Pain that has been so severe that it required seeking immediate medical attention

If you have answered no pain on question 18 you are now finished with the questionnaire.

If you have answered some type of pain the last month, please answer the following questions.
Grade the pain according to when you experienced the pain at its worst the last month:
0 = no pain, 10 = worst thinkable pain

19. At rest
20. When you take a 30m walk
21. When coughing
22. While walking in a staircase
23. Rising from a low chair
24. Pain in genital/inguinal area during sexual intercourse
25. How often have you experienced inguinal pain the last week?
    a. Once
    b. 2-5 times
    c. Every day
    d. Every day and during nights
    e. Continuous pain during day and night
26. Pain duration
    a. 1-60 minutes
    b. 1-5 hours
    c. All day
    d. All day and night
    e. Continuous pain during the week, day and night
27. Have you felt it necessary during the last week to take pain medication due to inguinal pain?
28. Has inguinal pain limited your possibilities to partake in recreational or sport activities?
    a. Yes, no, I don't normally partake in such activities.
29. Has inguinal pain limited your working ability?
    a. Yes, no, I don't have a physically demanding work.
30. To what extent has inguinal pain limited your working ability the last two months?
    a. I have not needed any sick leave due to inguinal pain.
    b. I have been on sick leave 1-7 days the last two months due to inguinal pain.
    c. I have been on sick leave 1-4 weeks the last two months due to inguinal pain.
    d. I have been on sick leave for the entire two months due to inguinal pain.
    e. I have received sick leave benefits due to inguinal pain.
    f. I do not have physically demanding work.

Preoperative Physician Questionnaire: Inguinal Hernia Related Symptoms
Date
Swedish social security number
Name
Side: Left, Right

1. Bilateral: yes, no
2. Palpable femoral hernia: yes, no, unsure
3. Palpable hernia with patient standing up: yes, no unsure
4. Is the hernia reducible? Yes, no.
5. Pain during palpation of inguinal canal?
6. Decreased or increased sensibility of light touch
    a. Inguinal region: yes, no
    b. Femoral region: yes, no
    c. Genitalia: yes, no
7. Pain at palpation over os pubis, hip, hip joint, muscle attachment areas or over inguinal ligament? Yes, no
8. Limitation or pain at flection, extension, abduction, adduction of hip? Yes, no
9. Pain at palpation of testis/spermatic cord (male), labia minora/majora (female)? Yes, no

Imaging for non-palpable suspected inguinal/femoral hernia:

10. Has ultrasound/CT/MRI been performed or is planned? Yes, no
11. If yes, is there a clear image of:
    a. Inguinal hernia.
    b. Femoral hernia.
    c. No hernia.
    d. Uncertain imaging.
12. Ultrasound shows a clear correlation between the hernia and the patients perceived pain/discomfort.
    a. Yes
    b. No
    c. Unclear – either no ultrasound was performed or the correlation between hernia and patient symptoms was not mentioned in the ultrasound report.

13. Do you assess that the patients' inguinal pain/discomfort is hernia related?
    a. Yes, no, unsure, the patient does not experience pain/discomfort.
14. What action is planned next?
    a. Referral to orthopedic surgeon.
    b. Active expectancy
    c. Hernia surgery
    d. Other investigation
15. Do you assess this patient has increased risk for chronic inguinal pain related to inguinal hernia surgery/mesh due to patient sex, age, pre-operative pain intensity or other pain symptoms? Yes, no.
16. Do you assess this patient has increased risk for chronic inguinal pain due to other pathologies not related to inguinal hernia surgery trauma (for example hip arthrosis)? Yes, no.

Patient postoperative questionnaire regarding inguinal pain and inguinal hernia surgery
Date
Swedish social security number
Name
In case of bilateral hernia, the questionnaire is filled out twice. This questionnaire is for: right, left

1. Following your inguinal hernia surgery have you had surgery in your lower abdomen/hip or other on the same side as your hernia? Yes, no.
    - a. If yes, which of the following:
        - i. Hernia recurrence
        - ii. C-section
        - iii. Prostate
        - iv. Sterilization
        - v. other
2. Do you perceive pain/discomfort in your thigh/hip on the same side as your hernia surgery during the last month? Yes, no
3. Do you have pain from other areas of your body for example prostate, uterus, muscle? Yes, no.
    - a. If yes, please specify.
4. Have you noticed a bulge in your inguinal region at some point in time during the last month? Yes, no.
5. Have you perceived genital discomfort on the same side as your hernia surgery at any time during the last month? Yes, no
    - a. If yes, pain or other
        - i. If other, please specify
6. In what way has the hernia surgery changed your inguinal pain/discomfort you perceived prior surgery
    - a. It is much better.
    - b. Somewhat better.
    - c. Unchanged.
    - d. Somewhat worse.
    - e. Significantly worsened.
7. Have you had any inguinal discomfort/pain on the side that has had surgery? Be aware this questionnaire is only for one side in case of bilateral hernia surgery. Yes, no.
    - a. If yes, pain or other.
        - i. If other, please specify.
8. Grade the inguinal pain in the on the side of surgical intervention when it was at its worst during the last week.
    - a. 1. No pain.
    - b. 2. Pain that can be ignored.
    - g. 3. pain that cannot be ignored but does not affect daily activities.
    - h. 4. Pain that cannot be ignored and that affects concentration/daily activities
    - i. 5. Pain that prevents performing most activities
    - j. 6. Pain that requires long periods of rest/bedrest
    - k. 7. Pain that has been so severe that it required seeking immediate medical attention

If you have answered 1. No pain on questions 7 and 8 you are now finished with the questionnaire.

If you have answered you perceive some kind of pain, please answer the following questions.

Grade the groin pain on the operated side when you experienced it at its worst during the last week.
0 = no pain, 10 = worst thinkable pain

9. At rest
10. When you take a 30m walk
11. When coughing
12. While walking in a staircase
13. Rising from a low chair
14. Pain in genital/inguinal area during sexual intercourse
15. How often have you experienced inguinal pain the last week?
    a. Once
    b. 2-5 times
    c. Every day
    d. Every day and during nights
    e. Continuous pain during day and night
16. Pain duration
    a. 1-60 minutes
    b. 1-5 hours
    c. All day
    d. All day and night
    e. Continuous pain during the week, day and night
17. Have you felt it necessary during the last week to take pain medication due to inguinal pain?
18. Has inguinal pain limited your possibilities to partake in recreational or sport activities?
    a. Yes, no, I don't normally partake in such activities.
19. Has inguinal pain limited your working ability?
    a. Yes, no, I don't have a physically demanding work.
20. To what extent has inguinal pain limited your working ability the last two months?
    a. I have not needed any sick leave due to inguinal pain.
    b. I have been on sick leave 1-7 days the last two months due to inguinal pain.
    c. I have been on sick leave 1-4 weeks the last two months due to inguinal pain.
    d. I have been on sick leave for the entire two months due to inguinal pain.
    e. I have received sick leave benefits due to inguinal pain.
    f. I do not have physically demanding work.

Thank you for your participation

Postoperative Physician Questionnaire: Inguinal Hernia Surgery Related Symptoms
Date
Swedish social security number
Name
Side: Left, right

1. Follow-up format: Phone consultation, outpatient consultation.
2. Palpable hernia with patient standing up? Yes, no, unsure.
3. Pain during palpation of inguinal canal? Yes, no.
4. Decreased or increased sensibility to light touch?
    a. Inguinal region: yes, no.
    b. Femoral region: yes, no.
    c. Genitalia: yes, no.
5. Pain at palpation over pubic bone, hip, hip joint, muscle attachment areas or over inguinal ligament? Yes, no.
6. Limitation or pain at flection, extension, abduction, adduction of hip? Yes, no.
7. Pain at palpation of testis/spermatic cord (male), labia minora/majora (female)? Yes, no.
8. Has ultrasound/CT/MRI been performed or is planned? Yes, no.
9. If yes, are there other probable causes to the patients' inguinal pain other than related to surgical trauma? Yes, no, uncertain.
    a. If yes or uncertain which possible causes could explain the patients pain?
        i. 1. Gynecological.
        ii. 2. Back.
        iii. 3. Hip.
        iv. 4. Muscle/tendon.
        v. 5. Internal medicine.
        vi. 6. Urological.
10. In case of telephone consultation, what was the reason the patient was not scheduled for a physical outpatient appointment?
    a. Social, economic or health issues.
    b. Discomfort/pain is not an issue.
    c. Incorrect filling of the questionnaire.
    d. The patient does not think there is any postoperative pain treatment.
    e. The patient perceives it is expected to experience postoperative discomfort/pain to some extent.
    f. The pain has improved/decreased.
    g. Other.
11. Has further investigation by other physicians found other probable cause to the patient's inguinal discomfort/pain other than that caused by surgical trauma (hernia surgery)?
    a. Yes, no, unsure, another referral was not necessary.
    b. If yes, what causes?
        i. Gynecological.
        ii. Back.
        iii. Hip.
        iv. Muscle/tendon.
        v. Internal medicine.
        vi. Urological.
12. Are there other causes of the patients' inguinal pain other than that related to surgical trauma? Yes, no, unsure, hernia recurrence.
13. Do you think the patients' inguinal pain is due to surgical trauma (i.e., not due to recurrence)? Yes, no, unsure.
14. What type of treatment was offered for chronic pain?
    a. Pharmacologic.

    b. Nerve block with local anesthetic.
    c. Surgery.
    d. Watchful waiting.
    e. Referral.
    f. Other.

15. Name the reason for why the patient did not receive pain treatment.
    a. Cannot/does not want it.
    b. The pain does not cause the patient that much trouble.
    c. The patient is wary of negative side effects of the offered pain treatment.
    d. The pain has improved/decreased.
    e. Other.
16. Has the treatment provided pain relief for the patient?
    a. Yes, more than 50% better.
    b. No.
    c. Partially, less than 50% better.
